# METRIC-EF: magnetic resonance enterography to predict disabling disease in newly diagnosed Crohn’s disease—protocol for a multicentre, non-randomised, single-arm, prospective study

**DOI:** 10.1136/bmjopen-2022-067265

**Published:** 2022-10-03

**Authors:** Shankar Kumar, Andrew Plumb, Sue Mallett, Gauraang Bhatnagar, Stuart Bloom, Caroline S Clarke, John Hamlin, Ailsa L Hart, Ilan Jacobs, Simon Travis, Roser Vega, Steve Halligan, Stuart Andrew Taylor

**Affiliations:** 1 Centre for Medical Imaging, University College London, London, UK; 2 Department of Gastroenterology, University College London Hospitals NHS Foundation Trust, London, UK; 3 Research Department of Primary Care and Population Health, University College London, London, UK; 4 Department of Gastroenterology, Leeds Teaching Hospitals NHS Trust, Leeds, UK; 5 Inflammatory Bowel Disease Unit, St Mark's Hospital and Academic Institute, London, UK; 6 Citibank, London, UK; 7 Kennedy Institute and Translational Gastroenterology Unit, University of Oxford and Biomedical Research Centre, Oxford, UK

**Keywords:** RADIOLOGY & IMAGING, Gastrointestinal imaging, Magnetic resonance imaging, Inflammatory bowel disease, Adult gastroenterology

## Abstract

**Introduction:**

Crohn’s disease (CD) is characterised by discontinuous, relapsing enteric inflammation. Instituting advanced therapies at an early stage to suppress inflammation aims to prevent future complications such as stricturing or penetrating disease, and subsequent surgical resection. Therapeutics are effective but associated with certain side-effects and relatively expensive. There is therefore an urgent need for robust methods to predict which newly diagnosed patients will develop disabling disease, to identify patients who are most likely to benefit from early, advanced therapies. We aim to determine if magnetic resonance enterography (MRE) features at diagnosis improve prediction of disabling CD within 5 years of diagnosis.

**Methods and analysis:**

We describe the protocol for a multicentre, non-randomised, single-arm, prospective study of adult patients with newly diagnosed CD. We will use patients already recruited to the METRIC study and extend their clinical follow-up, as well as a separate group of newly diagnosed patients who were not part of the METRIC trial (MRE within 3 months of diagnosis), to ensure an adequate sample size. Follow-up will extend for at least 4 years. The primary outcome is to evaluate the comparative predictive ability of prognostic models incorporating MRE severity scores (Magnetic resonance Enterography Global Score (MEGS), simplified MAgnetic Resonance Index of Activity (sMaRIA) and Lémann Index) versus models using standard characteristics alone to predict disabling CD (modified Beaugerie definition) within 5 years of new diagnosis.

**Ethics and dissemination:**

This study protocol achieved National Health Service Research Ethics Committee (NHS REC), London—Hampstead Research Ethics Committee approval (IRAS 217422). Our findings will be disseminated via conference presentations and peer-reviewed publications.

**Trial registration number:**

ISRCTN76899103.

**Strengths and limitations of this study**

- This is a multicentre, prospective study with a large sample size from National Health Service sites across the UK, all with established inflammatory bowel disease and magnetic resonance enterography (MRE) services.
- We exclusively study patients with a new diagnosis of Crohn’s disease with follow-up of at least 4 years.
- We evaluate the predictive ability of MRE using established scoring systems including the Magnetic resonance Enterography Global Score, simplified MAgnetic Resonance Index of Activity (sMaRIA) and the Lémann Index (LI).
- It is not possible to evaluate ultrasound in the present study as only static images, rather than cine loops will be available, and these will be of insufficient quality for analysis.

## Introduction

### Background and rationale

Crohn’s disease (CD) is a chronic, relapsing and remitting inflammatory disease of the gastrointestinal (GI) tract.^1 2^ Severity ranges from subtle mucosal ulceration to advanced transmural disease, which may be complicated by stricturing, fistulae and abscess.^3^ Some CD patients need regular hospital care,^4^ and 50%–80% require surgery.^5^ Imaging is crucial for diagnosis and staging because much of the small bowel (SB) is inaccessible to conventional endoscopy.^6–8^ Accordingly, at diagnosis patients with suspected CD undergo SB imaging as well as endoscopy.^9^ Magnetic resonance enterography (MRE) is increasingly used as the first-line imaging investigation in this scenario given its proven high accuracy for delineating disease distribution, severity and treatment response,^8 10–14^ while avoiding irradiation.^15 16^

Traditionally, CD treatment employs escalation of corticosteroids, immunomodulators and biological antitumour necrosis factor (TNF) in stepwise response to progressive symptoms.^6 17 18^ However, symptoms may not reflect underlying inflammation so a reactive approach risks irreversible bowel damage due to uncontrolled subclinical inflammation.^19^ The effect of tight control management on Crohn’s disease (CALM) trial demonstrated that CD treatment titrated to faecal calprotectin and blood C-reactive protein (CRP) resulted in superior bowel healing at 1 year compared with therapy based on symptoms alone.^20 21^ Accordingly, an alternative strategy that institutes advanced therapies early aims to prevent future complications such as strictures, penetrating disease, hospitalisation and surgery.

Advanced therapy usually employs biologics, such as anti-TNFα monoclonal antibodies, either alone or in combination with other immunomodulators.^2 9^ These agents are extremely effective at improving symptoms and healing bowel, but are associated with certain side-effects and are relatively expensive,.^22–24^ There is therefore a need for robust methods to identify patients who are most likely to benefit from early, advanced therapies and who will not. A systematic review and meta-analysis identified eight biomarkers displaying statistically significant prognostic potential to identify patients destined to develop severe/disabling Crohn’s disease. However, the review identified sparse primary research that evaluated cross-sectional imaging.^25^ The success of MRE as a staging and monitoring tool raises the possibility that it could also predict patient outcomes. While few series have explored a predictive role for MRE, these have not focused on newly diagnosed patients.^26 27^

Here, we describe the protocol for a non-randomised, single-arm, prospective study that aims to answer the question: ‘Do MRE features at diagnosis improve prediction of disabling CD within 5 years of diagnosis?’

### Objectives

#### Primary objective

- To improve prediction of disabling CD within 5 years of diagnosis by developing and internally evaluating a multivariable prediction model comprising both existing standard predictors and those based on MRE.

#### Secondary objectives

- To improve the prediction of disease phenotype within 5 years, defined by the Montreal behaviour criteria, by developing and internally evaluating a ‘baseline’ multivariable prediction model comprising standard clinicopathological variables.
- To identify specific combinations of individual MRE findings that best predict disabling CD within 5 years of diagnosis.
- To estimate the healthcare costs incurred within 5 years of a new diagnosis of CD and to explore patient, imaging and disease characteristics driving higher health economic costs.
- Assuming promising predictive potential, to then generate a research design for a subsequent prospective study to externally evaluate our MRE-based prediction model if appropriate.

### Study design

METRIC (Magnetic Resonance Enterography or Ultrasound In Crohn’s Disease) was a multicentre, prospective trial performed in eight National Health Service (NHS) centres across England and Scotland designed to compare the diagnostic accuracy of MRE and ultrasound (US) for the location and extent of CD.^15 28^ Consenting adult patients presenting with either newly diagnosed CD or presenting with suspected relapse were recruited: all underwent both MRE and US. Patients were followed up for 6 months minimum. In this study, we will draw solely on the group of patients who were recruited into METRIC with a new diagnosis of CD (ie, the ‘relapse cohort’ will be excluded). We will extend follow-up for the new diagnosis cohort to a minimum of 4 years.^15^ To achieve an adequate sample size, we will supplement newly diagnosed patients from METRIC (n=133) with a carefully matched retrospectively identified group of patients also newly diagnosed with CD, who did not participate in the METRIC trial.

## Methods

We adhered to the Standard Protocol Items: Recommendations for Interventional Trials reporting guidelines.^29^

### Study setting

The study will be conducted at nine UK NHS acute hospitals across England and Scotland, which participated in METRIC, supplemented by two additional NHS acute hospitals who will contribute retrospective accruals.^15 28^ One site from the METRIC trial is not included as they did not recruit any newly diagnosed patients.

### Eligibility criteria

**Figure 1:**
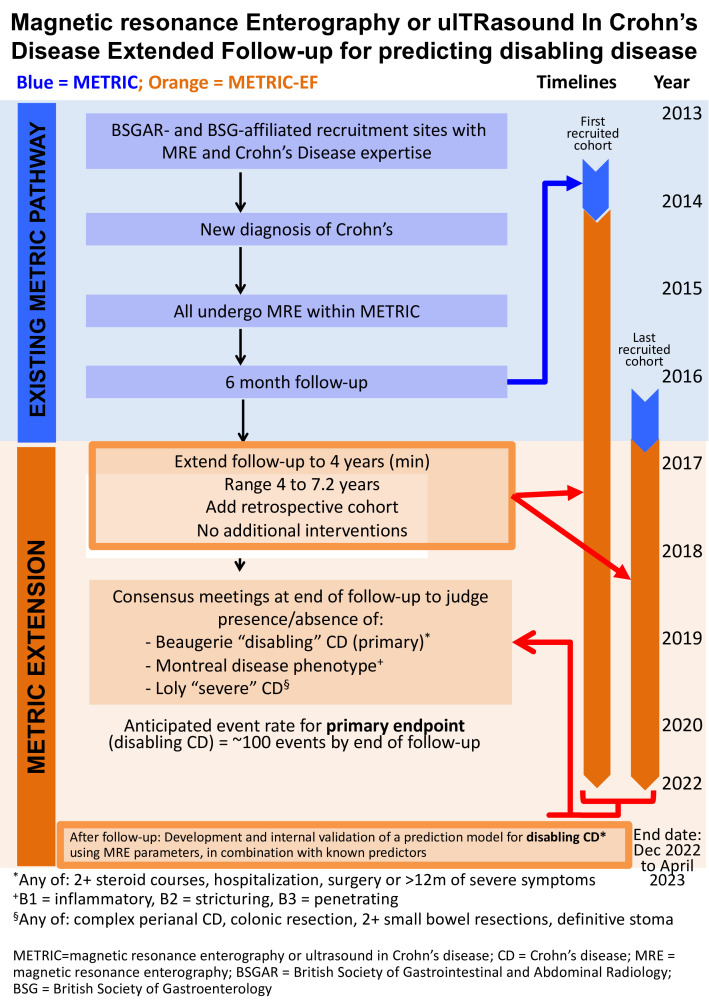
Flow diagram outlining the stages of the METRIC-EF trial. BSGAR, British Society of Gastrointestinal and Abdominal Radiology; BSG, British Society of Gastroenterology; CD, Crohn’s disease; MRE, magnetic resonance enterography; METRIC, magnetic resonance enterography or ultrasound in Crohn’s disease.

The study will focus on newly diagnosed CD, patients either (a) enrolled in METRIC (‘METRIC cohort’) or (b) imaged using MRE as part of their routine care at diagnosis (‘retrospective cohort’) (figure 1).

#### METRIC cohort: inclusion criteria

All confirmed new diagnoses from METRIC will be eligible for the present study; inclusion criteria therefore mirror those stipulated by METRIC:

- Patients aged 16 years or more.
- New CD diagnoses (within 3 months of time of recruitment), based on standard endoscopic, histological, clinical and radiological findings.

#### Additional retrospective cohort: inclusion criteria

The retrospective cohort will be added to METRIC accruals to achieve the required sample size.

Inclusion criteria for the retrospective cohort are as follows:

- Patients aged 16 years or more with newly diagnosed CD, based on endoscopic, histological, clinical and radiological findings.
- MRE acquired according to METRIC standard minimum sequence data set, and performed either <3 months before or after diagnosis.
- Normal institutional practice is to perform MRE in all new diagnoses of CD.
- At least 4 years clinical follow-up data available.

#### Exclusion criteria

Exclusion criteria for METRIC (and so carried forward) are as follows:

- Any psychiatric or other disorder likely to impact on informed consent.
- Evidence of severe comorbidities which makes it undesirable for the patient to participate in the study.
- Pregnancy.
- Contraindication to MRI (eg, cardiac pacemaker, severe claustrophobia and inability to lie flat).
- Final diagnosis other than CD.
- Enrolled in the METRIC study but not part of the final new diagnosis cohort.

### MRI

#### Sequences

METRIC recruits underwent a standard minimum MRE sequence data set (1.5 T or 3 T) (table 1). This will also be required for retrospective accruals, excepting diffusion-weighted imaging (DWI) which is unnecessary to calculate relevant activity and bowel damage indices.

**Table 1:** Required and optional sequences for the magnetic resonance enterography studies

Required	Optional
Coronal TrueFISP	Axial TrueFISP
Axial HASTE	Dynamic TrueFISP motility
Coronal HASTE	
Coronal HASTE with fat suppression	Axial HASTE with fat suppression
Axial DWI (b50 and b600)*	Additional b values
Coronal pregadolinium and postgadolinium VIBE (60–70s)*	Axial postgadolinium VIBE

*Optional for retrospective cohort.

DWI, diffusion-weighted imaging; HASTE, Half-Fourier Acquisition Single-shot Turbo spin Echo; TrueFISP, True Fast imaging with Steady State Precession; VIBE, volumetric interpolated breath-hold examination.

#### MRE activity scores

We will calculate established MRE activity indices as follows.

##### Magnetic resonance Enterography Global Score (MEGS)

This score encompasses aspects of both inflammatory activity and bowel damage, and has been validated against several reference standards, including a composite clinical reference,^30^ faecal calprotectin^31^ and capsule endoscopy^32^ (table 2).

**Table 2:** Calculation of Magnetic resonance Enterography Global Score (MEGS)

Mural features	0	1	2	3	Score
Mural thickness	<3 mm	>3–5 mm	>5–7 mm	>7 mm	a
Mural T2 signal (oedema)	Normal	Minor increase	Moderate increase	Large increase	b
Perimural T2 signal	Normal	Increased signal but no fluid	Small (≤2 mm) fluid rim	Large (>2 mm fluid rim)	c
Contrast enhancement: amount	Normal	Minor increase	Moderate increase	Large increase	d
Contrast enhancement: pattern	N/A or homogeneous	Mucosal	Layered		e
Haustral loss (colon only)	None	<1/3 segment	1/3–2/3 segment	>2/3 segment	f
Mural score for that segment					a+b+c+d+e+f =g
Multiplication factor	1	1.5	2	Total segmental score g * multiplication factor	
Length of disease in that segment	<5 cm	5–15 cm	>15 cm		

Each enteric segment (jejunum; proximal ileum; terminal ileum; caecum; ascending colon; transverse colon; descending colon; sigmoid colon; rectum) is scored separately. The segmental score is then multiplied by a factor depending on the length of disease involvement in that segment. Finally, scores for extramural features are added, giving a total score (maximum possible=296). Sum all segments, then add extramural score on a per-scan basis; five points for each of^1^: lymph nodes >1 cm short axis,^2^ comb sign (linear structures on the mesenteric border of an affected bowel segment),^3^ abscess and fistula^4^.

N/A, not applicable.

##### Simplified Magnetic Resonance Index of Activity (sMaRIA)

The simplified MAgnetic Resonance Index of Activity (sMaRIA) has been validated against endoscopic reference standards, and is used increasingly to assess treatment response in clinical trials^8 10–12 33^ (table 3).

**Table 3:** Derivation of the simplified MAgnetic Resonance Index of Activity (sMaRIA)

Feature	Description
Mural thickness	Binary: measured in mm using software callipers, scored as abnormal if >3 mm
Mural oedema	Binary: present if there is high signal intensity on T2 sequences with fat saturation, compared with normal-appearing loops
Fat stranding	Binary: present if there is loss of the normal sharp interface between the intestinal wall and mesentery, with oedema/fluid in the perienteric fat
Ulceration	Binary: present if mucosal surface has a deep depression, visible on 2 MRI sequences
sMaRIA score for that segment	=1 point for each of mural thickness, mural oedema and fat stranding; 2 points for ulceration (maximum 5 points per segment)

##### Lémann Index (LI)

The LI^34^ (table 4) is based on comprehensive assessment of structural bowel damage, including stricturing, penetrating lesions (ﬁstulae and abscesses) and surgical resection, and is applicable to different settings, such as early or advanced disease, patients with or without surgery, or with different CD locations and extension. The score comprises several factors that can be assessed either clinically, or using imaging or via endoscopy. We will use the imaging-derived score. Since the anal canal will not have been imaged specifically for METRIC, we will omit this score.

**Table 4:** Derivation of the Lémann Index

Organ	Method of assessment	N*	Segment	Grade 1	Grade 2	Grade 3
Surgical interventions†						
Upper tract	History	3	Oesophagus, stomach, duodenum	–	Bypass diversion or stricturoplasty	Resection
Small bowel	History	20	Each 20 cm SB segment	–	Bypass diversion or stricturoplasty	Resection
Colon/rectum	History	6	Each colonic segment	–	Stoma, bypass diversion or stricturoplasty	Resection
Stricturing lesions						
Upper tract	MRI	2	Stomach, duodenum	Wall <3 mm; segmental enhancement without prestenotic dilatation	Wall thickening ≥3 mm or mural stratification with no prestenotic dilatation	Stricture with prestenotic dilatation
Small bowel	MRI	20	Each 20 cm SB segment	Wall <3 mm; segmental enhancement without prestenotic dilatation	Wall thickening ≥3 mm or mural stratification with no prestenotic dilatation	Stricture with prestenotic dilatation
Colon/rectum	MRI	6	Each colonic segment	Wall <3 mm; segmental enhancement without prestenotic dilatation	Wall thickening ≥3 mm or mural stratification with no prestenotic dilatation	Stricture with prestenotic dilatation or >50% of the lumen
Penetrating lesions						
Upper tract	MRI	2	Stomach, duodenum	–	Deep transmural ulceration	Phlegmon or fistula
Small bowel	MRI	20	Each 20 cm SB segment	–	Deep transmural ulceration	Phlegmon or fistula
Colon/rectum	MRI	6	Each colonic segment	–	Transmural ulceration	Phlegmon or fistula

*n is the number of segments within a particular organ.

†This information will be collated from the original METRIC records, although a relevant surgical history will be very rare since included patients are, by definition, those with a new diagnosis of Crohn’s disease.

#### Interpretation and blinding

MRE scans will be interpreted by one from a pool of recruitment site radiologists; all are GI radiologists and experienced in MRE, in both clinical and research settings. Radiologists will be allocated MRE scans for scoring. These will be interpreted blinded to all clinical information other than that relevant for the calculation of the relevant index (eg, surgical history for LI).

#### Ultrasound

The arm from the METRIC trial will not be considered.

### Assessment of disease severity at follow-up

#### Time point of follow-up

Follow-up will be for a minimum of 4 years: since participants were recruited to METRIC over 30 months, this corresponds to an average follow-up of approximately 5.5 years. This provides sufficient time for clinically relevant complications of CD to manifest.^35–37^

#### Primary definition of disabling disease

The primary definition of disabling disease will be a modified version of that described by Beaugerie *et al*.^38^ The original definition has been modified to clarify some of the symptoms and to permit the use of disease-modifying therapy, since this has become a common preventative measure in modern practice. Disabling disease will therefore be defined as any of the following:

- Hospitalisation after CD diagnosis for flare or disease complication, as judged by the treating clinician.
- More than two corticosteroid courses required over 5 years and/or dependence on corticosteroids.
- Any intestinal resection >50 cm, or surgical operation for perianal disease (examination under anaesthesia without seton placement does not meet this criterion; abscess drainage and/or seton placement does).
- Chronic disabling symptoms, defined as a cumulative time of over 12 months of one or more of the following:
  - Diarrhoea with nocturnal stool (getting up for a bowel movement after having gone to bed).
  - Urgency (defined as having to rush to the toilet for a bowel movement).
  - Abdominal pain due to intestinal obstruction (requires imaging confirmation or surgical proof).
  - Fever (documented tympanic temperature of >38.0°C or oral temperature of>38.3°C).
  - Fatigue.
  - Joint pain not due to an alternative cause.
  - Uveitis.
  - Pyoderma gangrenosum.

#### Alternative definitions of disabling disease

Since the Beaugerie criteria are imperfect, further definitions of adverse outcomes will also be collected; specifically the Liège criteria^37^ and Montreal behaviour criteria.^39^

The Liège criteria are met if any of the following occur:

- Development of complex perianal disease.
- Any colonic resection.
- Two or more SB resections.
- A single SB resection of >50 cm.
- Definitive stoma.

Complex perianal disease is defined as per the American Gastroenterological Association,^40^ and the perianal disease modifier will be collected.

The Montreal behaviour criteria classify CD as either inflammatory (B1), stricturing (B2) or penetrating (B3). Stricturing disease will be defined as a fixed luminal narrowing of >50% relative to normal proximal bowel. Penetrating disease will be defined as an intra-abdominal or enterocutaneous fistula, inflammatory mass, or abscess.

#### Consensus panel assessment of disease severity

Consensus panels will be convened at each recruitment sites. Panels will comprise, as a minimum, one gastroenterologist and one radiologist, aided by the site research nurse. The consensus panels will review all available clinical information. This will include investigations such as CRP, faecal calprotectin, endoscopy (conventional and capsule), imaging (MRI, US, CT and fluoroscopy), surgical and histopathological findings, clinical activity scores (eg, Harvey-Bradshaw Index) and overall clinical course including outpatient and inpatient clinical records.

Using all the available data, the consensus panels will record the presence or absence of disabling disease, and Montreal classification according to the protocol definitions and the date at which this endpoint was reached.

### Outcomes

#### Primary outcome

Comparative predictive ability of prognostic models incorporating MRE severity scores (MEGS, sMaRIA and Lémann index) versus models using standard characteristics alone to predict disabling CD (modified Beaugerie definition) within 5 years of new diagnosis.

#### Secondary outcomes

- Comparative predictive ability of prognostic models incorporating MRE severity scores (MEGS, sMaRIA and Lémann index) versus models using standard characteristics alone to predict the development of Montreal B2 / B3 disease or Liège severe disease at 5 year follow-up.
- Identification of the best combination of individual MRE features to predict disabling CD within 5 years of new diagnosis.
- Average per-patient and national healthcare costs incurred within 5 years of new diagnosis.
- Patient, disease phenotype and imaging characteristics associated with higher economic costs within 5 years of diagnosis.

### Assumptions

We assume the prevalence of our modified Beaugerie definition of disabling disease will be approximately 55%–60%; this is informed primarily by the external validation cohort^37^ of the Beaugerie descriptors, in which 57% of 361 participants had developed disabling disease by 5 years. In support, a local audit of 33 newly diagnosed patients at one METRIC recruitment centre found 5 of 33 (15%) patients met the definition by mean 11.3 months, giving 16% at 1 year. Extrapolation to 5 years gives 58% prevalence, similar to that expected from the literature.^37^ We assume that development of disabling disease is approximately linear over time. Therefore, 207 participants will provide 114–124 events and 83–93 non-events (defined by non progression to disabling disease); the smaller proportion defines the minimum sample size for powering a modelling study, where regression methods are used for development (see section below).

### Sample size and justification

The sample size was based on including 207 participants newly diagnosed with CD. During the study, due to problems obtaining consent for additional follow-up hampered by COVID-19, the original target recruitment was necessarily reduced from 167 to 131 from the METRIC prospective cohort, and so an increased target of 76 participant retrospective cohort recruited from METRIC sites is anticipated. We anticipate this sample size will provide between 114 and 124 events (83–93 non-events). The number of the retrospective cohort will be increased to meet the 207 participant target if recruitment to the METRC cohort is below 131.

#### Adequacy of this number of events/non-events

Calculating sample sizes for prognostic studies suffers from a relative lack of readily applied methods suitable for all study designs, since sample size for development depends on whether the primary aim is to select potential variables for a new model (via univariable significance within a data set), or to evaluate a model where the variables have been prespecified and are therefore fixed. In the present study, the variables are fixed since we are explicit that we will test a small number of MRE scores in the context of a model using fixed clinical (?clinicopathological) variables. Therefore, recommendations for sample sizes relevant to external validation are most appropriate. Accordingly, the literature suggests we require 80–100 events for model evaluation where variables are prespecified and fixed.^41^ This also provides sufficient power to assess whether addition of the three MRE scores enhance prediction, under the widely used ‘rule-of-thumb’ of 10–20 events per variable.^42^ We note that recent methods to calculation external validation sample size did not exist in 2017, when this study was powered.^43^

### Power for secondary outcomes

#### Other definitions of adverse outcome

Development of Liège severe disease is estimated at 20% at 5 years.^37^ This provides approximately 41 events for the present study which is likely insufficient to develop meaningful prognostic models. Accordingly, analysis for this endpoint will be descriptive only, unless our assumptions prove incorrect and sufficient events satisfying this definition accumulate.

#### Identification of the most important MRE variables for model inclusion

Principal component analysis (PCA) will be used to reduce the number of individual MRE variables to ideally two or three eigenvector variables, for subsequent addition to the baseline clinical model. This will facilitate our ability to determine the effect on model fit of adding MRE variables.

### Retention

Participants need not undergo additional testing to enter this study. Only data obtained during routine clinical care are necessary to both define disabling disease and provide variables for model inclusion. Where participants are lost to local follow-up, participants’ general practitioner will be contacted to obtain routine clinical information, post consent (this is only applicable to METRIC cohort and those patients on retrospective cohort who have provided consent).

### Statistical methods—outcomes

#### Primary outcome

Comparative predictive ability of prognostic models incorporating MRE severity scores (MEGS, sMaRIA and Lémann index) versus models using standard characteristics alone to predict disabling CD (modified Beaugerie definition) within 5 years of new diagnosis

We will develop a multivariable prognostic model using prespecified standard predictors (age at recruitment as new diagnosis, smoking, gender, disease status at diagnosis (stricturing disease, perianal disease, severe endoscopic disease, location of disease as L1/L2/L3/L4, initial need for steroid therapy, weight loss of at least 5 kg, CRP, white cell count, faecal calprotectin, haemoglobin and platelets). Continuous variables will be retained where possible, with transformations and polynomial transformations when needed. Categorised variables will be retained as prespecified in clinical report form except where modelling requires combination of categories with small numbers. Missing covariates will be handled via multiple imputation, under the ‘missing at random’ assumption.^44^ We will compare the additive effect on model fit of each MRI score (MEGS, sMaRIA and LI as PCA variables) to the baseline standard model as a linear offset. An increase in model performance will be based on an improvement in the number of patients correctly classified for disabling disease, using models including MRE compared with a standard model. Model performance will be measured using sensitivity, specificity and net benefit. We will also assess difference in model fit using Bayesian Information Criteria (BIC) and we will report c-index for each model. Internal validation using bootstrap samples (sampling with replacement) will use at least 200 or more bootstrap samples until estimates remain stable. Model prediction at 1-year, 2-year and 3-year time horizons will also be reported.

#### Secondary outcomes

##### Secondary outcome 1

Comparative predictive ability of models incorporating MRI severity scores (MEGS, sMaRIA and LI) when compared with a baseline model of standard characteristics alone, for predicting Montreal B2/B3 disease or Liège severe disease in newly diagnosed patients by 5 years.

Modelling will be conducted as for the primary outcome. Models will only be developed if the number of events/non-events is adequate; if this is not achieved, only descriptive statistics will be provided.

##### Secondary outcome 2

Identification of the best combination of individual MRE features for prediction of disabling CD (all definitions) within 5 years of new diagnosis. PCA will be used to combine multiple MRE parameters into a small number of Eigenscore variables. This allows a larger number of features to be combined without compromising statistical power. The most influential imaging features will be identified for further simplification of MRE variables included in modelling. Methods will be as in the primary outcome, and the statistical significance of including MRE features will be evaluated based on improvement of model fit (BIC) in comparison to the standard model, with additional model performance reported as appropriate.

##### Secondary outcome 3

Average per-patient and national healthcare cumulative costs incurred within 5 years of newly diagnosed CD. Hospital healthcare usage from health economic case report forms (CRFs) will be multiplied by unit costs for relevant items, summed across the 5-year follow-up period, and averaged across the study population (median and mean). Mean costs per patient will be multiplied by the estimated number of CD patients in the UK, stratifying by the presence or absence of disabling disease, to estimate the cost-of-illness following a UK diagnosis (both by UK incidence and prevalence).

##### Secondary outcome 4

Patient, disease phenotype and imaging characteristics associated with higher economic costs, within 5 years of diagnosis. Unadjusted annual and 5-year costs will be calculated separately according to the presence or absence of disabling CD, Liège and Montreal criteria, MRE parameters, treatments received and patient demographics. Comparison between groups will be by one-way analysis of variance and Mann-Whitney two-sample tests. Multivariable regression will be used to identify factors (CD status, MRE parameters, treatments received and patient characteristics) associated with higher costs. To account for skewed cost data, we will use a generalised linear model with gamma family and log link, experimenting with other distributional assumptions (log-normal, Gaussian, inverse Gaussian and negative binomial distributions), selecting the best fit as judged by residual plots and the Akaike Information Criterion. A restricted version of the model will also be applied, only using data that are available at, and soon after, diagnosis.

### Economic evaluations

The health economic analysis will estimate healthcare costs incurred within 5 years of a new diagnosis of CD and investigate patient, imaging, treatment and other factors that drive these costs.

#### Health economic analysis

To estimate mean 5-year costs per patient, we require NHS hospital resource use data for all patients accumulated during the follow-up period. These will be collected in a similar manner to the METRIC study, which captured similar costs but only for a 6-month time horizon. A study-specific CRF will capture hospital resource use data for the following cost components for each patient during follow-up: all imaging investigations; endoscopy; surgery; outpatient visits; inpatient stays; day cases and medications. These will be populated at each site by the relevant research team. Unit costs will be obtained from standard published sources, including NHS tariffs.

### Ethics and dissemination

#### Consent

The new diagnosis cohort patient recruited to METRIC will be approached and consented (if willing) for participation in METRIC-EF. Patients refusing participation will be excluded. We have been granted permission to collate data from the retrospective cohort without direct patient consent as there is no direct patient intervention and pseudonymised data only will collected by the Clinical Trial Unit.

#### Ethical permission

The METRIC-EF study achieved National Health Service Research Ethics Committee (NHS REC), London—Hampstead Research Ethics Committee approval on 26 October 2018 (IRAS 217422) and is being conducted in accordance with the principles of Good Clinical Practice. Informed consent is a requirement. University College London’s Clinical Trials Unit is supervising the study.

#### Patient and public involvement

Our patient representative will ensure dissemination to patient groups via Crohn’s and Colitis UK.

#### Dissemination plans

Data will be pseudonymous during the study; only fully anonymised data will be published, without any identifiers. Consented participants will be informed of the study results during outpatient follow-up appointments.

## Discussion

METRIC-EF is a multicentre, non-randomised, single-arm, prospective study of adult patients with newly diagnosed CD. It aims to determine if MRE features at diagnosis improve prediction of disabling CD within 5 years of diagnosis. Accurate prediction of a disabling disease trajectory would have major implications by facilitating identification of patients most likely to benefit from early, advanced therapies, while simultaneously avoiding unnecessary treatment and costs in others. We will enrol patients already recruited to METRIC and extend their follow-up,^15^ supplemented by a separate retrospective cohort to achieve adequate sample size. It is not possible to evaluate US in the present study as only static images, rather than cine loops were returned by most sites, which were of insufficient quality for analysis.

We believe this is the first study to investigate MRI as a predictive biomarker for development of disabling disease in newly diagnosed CD. In a dual-centre prospective study of 142 CD patients, Fiorino *et al* evaluated the predictive role of MRE on disease outcome and found that bowel damage on imaging was associated with increased future hospitalisation and surgery.^27^ However, patients were eligible if imaging was acquired within 2 years of a potential diagnosis of CD, so was not representative of a newly diagnosed cohort. A single-centre study enrolled 112 patients with relapsed CD (rather than new diagnoses) and conducted both MRE and colonoscopy.^26^ Future surgical resection was related to the presence and degree of established bowel damage (stricture, fistula or abscess) rather than the degree of inflammation. Most recently, a single-centre study of 52 patients with CD (not stratified by new diagnosis/suspected relapse) found that the presence of restricted diffusion, increased upstream dilatation from a stricture, complex fistula, perienteric inflammation, fibrofatty proliferation and increased length of disease involvement on outpatient MRE were significantly more common in patients progressing to surgery.^45^ It is unknown if these findings can be extrapolated to new diagnoses, who, by definition, are earlier in their disease trajectory than those with relapsed, established CD.^26^ Nevertheless, these studies suggest that the degree of established bowel damage may predict future adverse outcomes, rather than the degree of inflammation encountered during a flare. Unlike other biomarkers such as CRP and calprotectin, MRE has the advantage of being able to quantify both bowl damage and inflammation simultaneously.

### Trial status

Trial recruitment began in 2018 but has been significantly delayed due to the COVID-19 pandemic. We anticipate closure of the study either during the final quarter of 2022 or first quarter of 2023.

## Supplementary Material

Reviewer comments

## Data Availability

No data are available.
