# Supplementary material for: METRIC-EF: magnetic resonance enterography to predict disabling disease in newly diagnosed Crohn’s disease—protocol for a multicentre, non-randomised, single-arm, prospective study
Source: BMJ Open. 2022 Oct 3;12(10):e067265. doi: 10.1136/bmjopen-2022-067265 (PMC9535152; doi:10.1136/bmjopen-2022-067265)
Supplement: Reviewer comments [file bmjopen-2022-067265.reviewer_comments.pdf]

PEER REVIEW HISTORY

BMJ Open publishes all reviews undertaken for accepted manuscripts. Reviewers are asked to complete a checklist review form (**http://bmjopen.bmj.com/site/about/resources/checklist.pdf**) and are provided with free text boxes to elaborate on their assessment. These free text comments are reproduced below.

ARTICLE DETAILS

**TITLE (PROVISIONAL)**	METRIC-EF: Magnetic Resonance Enterography to predict disabling disease in newly diagnosed Crohn's disease – Protocol for a multicentre, non-randomised, single arm, prospective study
**AUTHORS**	Kumar, Shankar; Plumb, Andrew; Mallett, Sue; Bhatnagar, Gauraang; Bloom, Stuart; Clarke, Caroline; Hamlin, John; Hart, Ailsa L.; Jacobs, Ilan; Travis, Simon; Vega, Roser; Halligan, Steve; Taylor, Stuart

This article was not externally reviewed at BMJ Open. Protocols that have been independently assessed prior to submission to BMJ Open are usually fast-tracked to publication on the grounds that further substantial changes will not be possible. This independent assessment will usually be external, independent review for both a substantial grant award from a non-commercial or government funder and ethics approval.
